# Progress in Surgery

**Published:** 1897-03-20

**Authors:** 


### PROGRESS IN SURGERY.

### DISEASES OF THE LARYNX, &c.

(*Continued from page* 399.)

**Laryngeal Tuberculosis.**—Solomon S. Cohen[15] reports very favourable results from the local use of formic compounds, *e.g.*, iodoform, bromoform, and formaldehyde. The latter he employs in solutions of formalin in strength from one to ten per cent., applied after cocainisation. He has been able to get tuberculous lesions to heal under its use. Bromoform he uses chiefly as a local analgesic, while with guaiacol locally applied he has had a favourable but less extensive experience. Hedderich has used para-mono-chlor-phenol in laryngeal phthisis with favourable results, but Lubinski after trying it gave it up, as it had so many disadvantages.

**Ludwig's Angina.**—It is interesting to note the relatively frequent instances recorded which support the view propounded by Semon as to the pathological (as distinguished from bacteriological) identity of various septic processes clinically termed Ludwig's angina, Senator's phlegmon, erysipelas, &c. J. Bobone[16] relates a case of Ludwig's angina in a man aged twenty-eight, who had been suffering from chronic urethral blenorrhagia for several years, with exacerbations. All the local and general symptoms of angina Ludovici developed, and creamy fœtid pus was let out by incision of the swelling. The author was able to exclude dental caries, malarial infection, and influence of cold as direct causes in this case, and as the patient was suffering from an acute attack of gonorrhœa, was inclined to attribute the angina to direct gonorrhœal infection, although he was unable to find the gonococcus in the pus.

**Diphtheria.**—The limitations of space preclude anything like a reference to all the statistics of cases treated by anti-toxic serum, but we may refer to one of the most important and elaborate collections of cases recently recorded, the American Pediatric Society's collective investigation[17] into the use of antitoxin in the treatment of diphtheria in private practice. Reports from 615 different physicians, with 3,628 cases, were returned. Of these 244 cases were excluded from their tables, these being cases in which the disease was confined to the tonsils and unconfirmed by cultures. Thus only cases in which the diagnoses were confirmed by culture, and others giving pretty clear evidence of diphtheria, either in the fact that they had been contracted from other undoubted cases, or where the membrane had invaded other parts besides the tonsils, such as the palate, pharynx, nose, or larynx, are included. This is the important point, that the 3,384 cases analysed were almost certainly cases of true diphtheria. Other facts enhance the value of the figures, inasmuch as in many cases the reporters stated that the serum was only resorted to when the condition of the patient had become alarmingly worse under ordinary methods of treatment, statements confirmed by the unusually large number of cases which were injected late in the disease. Again, many physicians, dreading some unfavourable effects of the serum, hesitated to use it in mild cases, and only gave it in those cases which from the outset gave evidence of being of a severe type. The expense, too, unquestionably deterred many from employing the serum in mild cases. These facts, the committee state, should more than outweigh the bias of any anti-toxin enthusiasts by including many mild cases which would have recovered under any treatment. We have alluded in some detail to the preliminary remarks in the report, because several clinicians, who seem unable to read figures aright, have endeavoured to blind us to the obvious conclusion that the anti-toxin treatment has had a potent influence on the course of the disease.

The report also includes 942 cases treated by the New York Health Board, every one of these except 26, and in these 26 the diagnosis rested on extensive deposit of membrane or laryngeal invasion. These 942 cases were of a more than ordinarily severe type, more than 50 per cent. of them being reported as in bad condition at the time of injection; to mild cases the inspectors were not often called.

Taking the whole of the 4,326 cases we get the following results:—

| | Injected 1st day. | 2nd day. | 3rd day. | 4th day. | 5th day. | Day unkn. | Total. |
|---|---|---|---|---|---|---|---|
| Committee's report ... | Mort. 4·9% | 8·3% | 12·7% | 22·9% | 38·9% | 7·0% | 13·0% |
| New York Health Bd. | Mort. 8·7% | 12·0% | 16·6% | 20·9% | 29·0% | 23·5% | 17·8% |

TABLE II.—AGE AND RESULT OF TREATMENT.

| | 0 to 2 years. | 2-5 years. | 5-10 years. | 10-15 years. | 15-20 years. | 20 yrs. & over. |
|---|---|---|---|---|---|---|
| Committee's report ... ... | Mort. 21·7% | 13·7% | 12·2% | 6·8% | 3·6% | 4·2% |
| New York Health ... | Mort. 27·5% | 17·8% | 11·2% | 0·0% | 0·0% | 0·0% |
| Total ... | 23·3% | 14·7% | 12·1% | 6·2% | 3·2% | 3·8% |
| Excluding moribund cases ... | Mort. 19·2% | 13·3% | 8·7% | 3·3% | 3·2% | 2·1% |

Referring in their summary to the laryngeal cases, the report states that the most convincing argument, and, to the minds of the committee, an absolutely unanswerable one, in favour of serum therapy is found in the results obtained in the 1,256 laryngeal cases (membranous croup). In one half of these recovery took place without operation, in a large proportion of which the symptoms of stenosis were severe. Of the 533 cases in which intubation was performed the mortality was 25·9 per cent., or less than half as great as has ever been reported by any other method of treatment.

**Tuberculosis of the Tonsils.**—Ruge[18] stated that tuberculous disease of the tonsils is more common than is generally believed. He relates a case of his in which the tonsillar disease was primary. Not till a year after the tonsil began to enlarge was there any other evidence of disease elsewhere. He also examined the tonsils microscopically in 17 other cases, and in seven there were evidences of tuberculous disease, five of these having pulmonary tuberculosis. He asserts that infection may be brought about (1) by the blood (as in miliary tuberculosis); (2) by lymph; (3) by the expectoration; (4) by air; (5) by food.

[15] N. Y. Med. Jour., Oct. 24, 1896. [16] Bollet delle Malat. dell' Orrechio, Agosto, 1896; Jour. of Laryng., Feb., 1897. [17] American Jour. of Pediatrics, Supplement. [18] Virchow's Arch. Bd., cxliv., June, 1896; Med. Chron., Dec., 1896.

### GENITO-URINARY SURGERY.

(*Continued from page 400.*)

**Stone Impacted in the Ureter.**—Jordan Lloyd refers[12] to the similarity in the symptoms of stone impacted in the ureter and stone in the kidney. In all his cases of ureteral stone a diagnosis of renal calculus had been made. When situated close to the opening into the bladder the symptoms resemble those of vesical stone. The diagnosis of impaction of a stone in the ureter he considers very difficult, but suggests that if symptoms of renal calculus are present, but there is an absence of pricking or stabbing pain on palpation or prodding the kidney, the stone is more likely to be in the ureter, especially if there is tenderness on deep pressure at any point along its course. When the stone lies in the lower end of the ureter it may sometimes be felt from the rectum or vagina. He thinks stone impacted in the upper half of the ureter can be most easily and safely removed through a lumbar incision; stone in the ower half of the duct through an extra peritoneal liliac incision, exactly like that generally employed for ligature of the common iliac artery; and stone impacted in the opening of the ureter into the bladder, or lying in that part of the duct which traverses the bladder wall, could be most readily reached through a suprapubic vesical incision. He suggests that it might be possible to remove a stone in this situation, in the male, through a perineal incision, such as has been employed for extirpation of a seminal vesical, or in the female through a forcibly dilated urethra, or through an incision made transversely in the lateral roof of the vagina into the space between the layers of the broad ligament. He records a case in which the symptoms resembled those of renal stone. On exploration of the kidney a full-length bougie, passed down the ureter, failed to discover the stone, but it was found by intraperitoneal incision, and extracted by the extraperitoneal route. The incision in the ureter was not sutured, but no urine escaped, and rapid and perfect recovery took place. Israel relates[13] three cases of ureteral calculus, and refers to the difficulty of diagnosis. He concludes that in anuria due to an impacted stone an operation undertaken even in coma may save life, but that it is much more likely to be successful if undertaken early. In such cases delay should not extend over 24 to 48 hours. The apparently satisfactory state of the patient should not be allowed to mislead.

**Valvular Stricture of the Ureter.**—Fenger, of Chicago, calls attention to the existence of these strictures, and describes an operation which he has practised for their removal.[14] He says that in cases of hydronephrosis or pyelonephrosis it is not uncommon to find comparatively narrow or thin-walled semilunar valves lying transversely in the ureter, and opening upwards. They have the same mechanical action as valves in veins, and obstruct the passage of urine along the duct. Sometimes there are two or three in one ureter. By causing a gradually increasing impediment to the flow of urine they produce dilatation of the ureter above, and usually near to, the valve, so that a ureter with many valves will have several dilatations, and stones may lodge in these. These valves can be removed by a longitudinal incision in the ureter, and Fenger considers this operation preferable to Van Hook's method of excising the entire wall of the ureter, with invagination of the divided ends.

**Bilocular Intrapelvic and Scrotal Hydroceles** are described[15] by MacEwen, of Glasgow. They form hydroceles in the scrotum which pass through the ring like a hernia; and the scrotal portion is continuous with an intrapelvic sac which extends along the pelvic portion of the cord. In one of MacEwen's cases this pelvic portion of the sac pressed on the bladder, and gave rise to great frequency of micturition. He easily dissected out the deeper portion of the hydrocele. In a case recorded by M. Bazy, a marked abdominal swelling continuous with the scrotal one, and extending above the umbilicus, was present. In another of MacEwen's cases, the pelvic portion of the hydrocele could be readily felt from the rectum, and fluctuation could be obtained through to the scrotal portion.

[12] Brit. Med. J., Oct. 24, 1896. [13] Berl. Clin. Woch, Sept. 21, 1896; and Epit B..M.J., Oct. 24. [14] Am. J. of Med. Sciences, Dec., 1896. [15] Practioner, Aug., 1896.

